# Effects of dietary supplementation of bacteriophage cocktail on health status of weanling pigs in a non-sanitary environment

**DOI:** 10.1186/s40104-023-00869-6

**Published:** 2023-05-08

**Authors:** YoHan Choi, Abdolreza Hosseindoust, Sang Hun Ha, Joeun Kim, YeJin Min, YongDae Jeong, JunYoung Mun, SooJin Sa, JinSoo Kim

**Affiliations:** 1 Swine Science Division, National Institute of Animal Science, Rural Development Administration, Cheonan, 31000 Republic of Korea; 2 Department of Animal Industry Convergence, Kangwon National University, Chuncheon, 24341 Republic of Korea

**Keywords:** Antioxidant, Cytokines, Diarrhea, Fecal score, Inflammation, Microbiota, Proteobacteria

## Abstract

**Background:**

The study evaluated the effects of bacteriophage cocktail (BP) and ZnO administered during weaning time for piglets exposed to a non-sanitary environment. The bacteriophages were designed to eliminate *Escherichia coli* (K88, K99 and F41), *Salmonella* (*typhimurium* and *enteritidis*), and *Clostridium perfreingens* (types A and C). Forty 21-day-old crossbreed piglets were assigned to four treatments, including the PC (sanitary environment), NC (non-sanitary environment), BP (NC plus 10^8^ pfu/kg BP), and ZO (NC plus 2,500 mg/kg ZnO). Piglets in the NC, BP and ZO were kept in a non-sanitary environment for 14 d, which was contaminated with the feces of infected pigs.

**Results:**

Pigs in the BP and ZO treatments had a higher final body weight compared with the NC. The NC treatment showed the highest concentration of inflammatory cytokines including interleukin (IL)-1β, IL-6 and tumor necrosis factor-α in the plasma. The administration of BP and ZO showed lower myeloperoxidase concentrations compared with the NC. The NC treatment showed a lower concentration of superoxide dismutase in serum compared with the PC. Among the treatments in non-sanitary environment, the NC treatment showed a higher concentration of malondialdehyde compared with the ZO. The PC treatment showed a lower concentration of butyric acid in the feces compared with the BP treatment. Among non-sanitary treatments, the villus height in the duodenum was greater in the BP and ZO compared with the NC. The lower abundance of Proteobacteria phylum was observed in the BP and PC treatments compared with the NC. The highest relative abundance of *Eubacterium* was recorded in the BP treatment. The abundance of *Megasphaera* and *Schwartzia* was higher in the NC pigs compared with the BP piglets. The abundance of *Desulfovibrio* was lower in the supplemented treatments (BP and ZO) compared with non-supplemented (NC and PC). The abundance of *Cellulosilyticum* genera was higher in the BP and ZO treatments rather than in the NC. The piglets in the NC treatment had the highest abundance of *Escherichia-Shigella*, followed by the PC and ZO treatments.

**Conclusion:**

In conclusion, these results suggest that the supplementation of bacteriophage cocktail could effectively control Proteobacteria phylum, *Clostridium* spp. and coliforms population and mitigated the adverse influences of weaning stress in piglets.

## Introduction

Post-weaning diarrhea remains a critical disease mostly caused by *Escherichia coli* and *Clostridium* spp. with high economic loss during the weaning period [1]. During the weaning period, pigs deal with nutritional, environmental and psychological stresses [2–4]. These challenges enforce pigs to be susceptible to intestinal microbial changes and pathogenic diseases. The release of enterotoxins, heat-labile enterotoxins, and lipopolysaccharide (LPS) to the intestine are stressful traits during diarrhea incidence [5]. The F4 fimbria is a type of bacteriophage-encoded pili that is involved in the adhesion of the pathogenic *Escherichia coli* to host cells [3]. This mechanism can play a role in the colonization and virulence of the bacteria, leading to diarrhea [3, 6]. The F4 fimbria-mediated (K88^+^ and K99) are the most common strains that initiate the colonization in the intestinal brush border connecting anchors to the F4 receptors [3]. These reactions increase diarrhea incidence and induce inflammatory responses [7, 8]. Intestinal inflammation and poor growth performance are linked to imbalances in the gut microbiota caused by pathogenic colonization during the weaning period [8]. It can be more of an issue when pigs grow in non-sanitary conditions, which is a common issue, particularly in small-scale farms. Poor cleaning and quarantine procedures allow transferring pathogens from infected pigs to the subsequently accommodated pigs [1].

Farms with poor sanitation, require full attention by supplementing preventive anti-pathogenic items to control mortality and morbidity, particularly if the previous pigs were severely infected. Increasing the persistence of weaned pigs against *E. coli* and *Clostridium* spp. will substantially decrease diarrhea and economic losses due to reduced transmission [6, 9–11]. Providing in-feed supplements to piglets diet during weaning is a preventive practice that presents opportunities for controlling diarrhea [12–15]. There have been numerous strategies to attenuate the adverse effects of diarrhea by non-antibiotic additives, especially in a non-sanitary environment [16, 17]. A pharmacological dose of ZnO has long been added to the weaned diet as the most practical and effective method in the past two decades. ZnO properly reduced the tendency to increase recurrent diarrhea and intestinal injuries in pigs [16, 18]. However, the use of ZnO has been phased out since June 2022, due to high Zn excretion and consequent environmental issues [15, 19]. Regarding the recent legislated rules, there is a growing interest in finding alternatives with comparable growth effects and costs with long-term benefits. Recently, bacteriophages, are being increasingly used as an effective alternative to eliminate pathogens and promote intestinal health in weaned pigs [8, 20, 21]. Bacteriophages have the preference of eliminating only some special species of pathogen rather than having non-selective effects on microbiota like antibiotics [16, 17, 22, 23]. Therefore, the selectivity of bacteriophages in eliminating the targeted pathogens may be considered a positive factor to be used in pigs in a non-sanitary environment. Moreover, there is a possibility of supplementing bacteriophages for a longer period from before weaning to the time of diarrhea incidence in order to control diarrhea at the beginning stages [21, 24], as well as prevent the horizontal spread of *E. coli* and *Clostridium* spp. among pigs. With advances in production technology, the recent bacteriophages are semi-pH stable with the capability of surviving and multiplying in the intestinal environment. There are several reports on the role of bacteriophage in eliminating pathogens in farm animals in a controlled environment [1, 21, 25]. However, no study is available on the role of bacteriophage cocktails in preventing pathogens in a non-sanitary environment, which might be more similar to a farm scale. Our study was performed to evaluate the influences of bacteriophage cocktail on the pathogen growth control, immune system and fecal microbial changes of weaned pigs in a non-sanitary environment.

## Materials and methods

### Animals, experimental design, and diets

This experiment was conducted at the facility of National Institute of Animal Science Farm in the Republic of Korea. The farm staff frequently carried out standard farm management and husbandry procedures. Weaned crossbred piglets weighing 7.18 ± 0.41 kg (Duroc × Yorkshire × Landrace) were put into four groups (*n* = 10 for each treatment). Water and food were available to the piglets ad libitum. The treatments included the PC (sanitary environment), NC (non-sanitary environment), BP (NC plus 1 × 10^8^ pfu/kg bacteriophage cocktail), and ZO (NC plus 2,500 mg/kg ZnO).

### Animal feeding, management and non-sanitary environment protocol

The experiment was conducted for 14 d. On the first day of the experiment and the last day (d 14), each experimental animal was weighed separately in order to determine body weight (BW) and average daily gain (ADG). Piglets were sacrificed using an approved anesthetic at the end of the experiment, and tissue and digesta samples were taken right away after exsanguination. Weaned diarrheal pigs were introduced to the experimental room 14 d prior to the start of the experiment in a contaminated environment. The slurry pit of the contaminated room was washed and the manure was superficially cleaned from the cages, but no disinfectant was used. Twenty-five bacterial samples were collected from feeders, cage walls and floors before placing the pigs in order to assess the level of bacterial contamination.

### Preparation of bacteriophages

The bacteriophage cocktail included bacteriophages targeting *E. coli* (K88, K99 and F41), *Salmonella* with antibacterial activity against *S. typhimurium* and *S. enteritidis,* one *Clostridium perfringens* bacteriophage with antibacterial activity against *C. perfringens* types A and C (CTCBio, Inc., Seoul, Korea). The individual bacteriophage was isolated utilizing a succession of isolation procedures from a variety of samples, including water, soil and farm waste (plaque isolation, co-cultivation with host strains, centrifugation, and filtration). Bacteriophages with bacteriolytic activity against *E. coli* K88, K99, F41*, S. typhimurium, S. enteritidis,* and *C. perfringens* (types A and C) were isolated to be cultured. By combining individual powdered bacteriophages that had been synthesized as follows, the bacteriophage cocktail was created. The bacteriophage and log-phase host strain were co-cultivated in tryptic soy broth (Difco, Detroit, MI, US), until the lysis of the host bacterial cell was visible. After centrifuging the culture at 10,000 × *g* for 30 min at 4 °C, the resultant supernatant was purified with a 0.45-µm syringe filter. Through increasing the bacteriophage titre, the entire process from co-cultivation to filtration was carried out twice. After adding maltodextrin (50%, w/v), and pulverizing with a mill, the obtained filtrates containing individual bacteriophages were freeze-dried. The bacteriophage was produced using the individual bacteriophage's powdered byproduct. After being combined with commercial complete feed at a weight ratio of 0.1%, the bacteriophage was fed to weaned piglets. The content of bacteriophage in the bacteriophage product was adjusted to be approximately above 10^7^ plaque-forming units per gram (pfu/g) for *E. coli* K88 (8 × 10^6^), K99 (1.2 × 10^6^), F41(2.1 × 10^6^), 10^8^ pfu/g for *S. typhimurium* (4.2 × 10^7^)*, S. enteritidis* (6 × 10^7^)*,* and 10^7^ pfu/g for *C. perfringens* (types A and C).

### Fecal score

The incidence of diarrhea was measured by scoring the feces as 0 (normal), point 1 (normal feces), point 2 (moist feces), point 3 (mild diarrhea), point 4 (severe diarrhea), and point 5 (watery diarrhea) in all the experiments. The overall cumulative incidence of diarrhea was measured daily at 0900 h on d 1, 3, 5, 8, 11 and 14, and the final diarrhea incidence was determined as the average of the scores. Following observations of individual pigs and indications of feces consistency, scores were recorded for each pen.

### Short-chain fatty acids

A fresh fecal sample was taken by rectum massage on d 14 and kept at −80 °C until the completion of the collection period before being sent immediately to the lab. According to Oh et al. [26], a sample of approximately 1 g of feces was taken, weighed and diluted with 2 mL of deionized water. The sample was then centrifuged at 10,000 × *g* (4 °C) for 20 min to form a supernatant. Then, a sample of 25% metaphosphoric acid solution was combined in a 9:1 ratio and centrifuged at 3,000 × *g* for 10 min. A 0.45-mm filter membrane was used to filter the supernatant after it was aspirated with a syringe. The YL 6500 gas chromatography (Gyeonggi-do, Korea) including TRB-G43 capillary column with inner diameter of 0.53 mm and 30 m length, and 3 μm film thickness, outfitted with a flame ionization detector, was used to analyze acetate, propionate, butyrate and total short-chain fatty acids (SCFA). After 3 min, the column's temperature rose to 150 °C from a starting point of 70 °C. Each injection volume was 1 μL, and the temperature of the injector and detector was 250 °C.

### Intestinal histomorphology

For the intestinal histomorphology study, mucosal and histological tissue samples were taken from the duodenum, jejunum and ileum. The remaining samples were then frozen in liquid nitrogen and kept at −80 °C. The samples from the duodenum, jejunum and ileum were cut into sections of about 5 cm, neutral buffered 10% formalin was used to fix them for 24 h, and then they were transferred to a 70% ethanol solution, wax-embedded and stained with hematoxylin and eosin. Each slice was put on a slide for analysis as previously reported [27]. Five well-characterized villi and crypts from each segment were counted to measure intestinal morphology. Both the crypt depth (CD) measured from the villi base at the lowest point of the CD and the villus height (VH) measured from the villi tips up to the villi-crypt junction were recorded. The Vanox-S Microscope (Olympus Corporation, Lake Success, NY, US) was used to evaluate intestinal sample slide evaluation, and SPOT simple imaging software was used to analyze the images (Diagnostic Instruments, Sterling Heights, MI, US).

### Cytokines and zonulin concentration in serum

At the end of trial (14 d), all pigs were subjected to the analysis for serum cytokines and zonulin. Blood sample was harvested from the jugular vein. For the purpose of separating the plasma, collected blood was transferred into untreated vacuum tubes and heated to 25 °C. The vacuum tubes were then centrifuged at 2,500 × *g* for 10 min. Interleukin (IL)-6 (Cat # MBS742458, MyBioSource, San Diego, CA, US), IL-1β (Cat # MBS700738, MyBioSource, San Diego, CA, US), tumor necrosis factor-α (TNF-α) (Cat # MBS2019932, MyBioSource, San Diego, CA, US), and zonulin (Cat # MBS2607498, MyBioSource, San Diego, CA, US) levels in serum were measured after it had been aspirated into a 2.5-mL centrifuge tube and kept at −20 °C.

### Inflammation-related enzymes in the jejunum

The jejunum samples (50 mg) were immediately harvested after euthanization from the distal section of jejunum. The tissue samples were homogenized with a cold buffer (pH 7.7 containing 1 mmol/L EDTA; 0.1 mmol/L Tris–HCl), then centrifuged at 8,000 × *g* for 20 min at 4 °C after sonicating for 20 s. The concentrations of myeloperoxidase (MPO), inducible nitric oxide synthase (iNOS), and cyclooxygenase-2 (COX-2) in jejunum were determined according to the manufacturer’s manuals using ELISA kits (MPO, #MBS700708; iNOS, #MBS901536; COX-2; #MBS026345; MyBioSource, San Diego, CA, US).

### Antioxidant indicators in serum

According to Hosseindoust et al. [28], the samples were pre-treated and evaluated using a MyBioSource kit manual in order to assay the antioxidant factors activity in serum (Enzyme activity assay, MyBioSource, San Diego, CA, US). Malondialdehyde (MDA; Cat #MBS2801692, MyBioSource, San Diego, CA, US) and superoxide dismutase (SOD; Cat # MBS040378, MyBioSource, San Diego, CA, US) concentrations were assessed in the serum samples that had been taken. According to the MyBioSource kit's instruction manual, a microplate reader (Power Wave XS, BIoTeK, Winooski, VT, US) was used to assess absorption detection. The levels of aspartate aminotransferase (AST) and alkaline phosphatase (ALP) were evaluated using an automatic spectrophotometer (Roche, Branchburg, NJ, US).

### DNA extraction and 16S rRNA amplification and sequencing

Following the manufacturer's instructions, genomic DNA was extracted from 300 mg of each fecal sample using a NucleoSpin Soil kit (Macherey–Nagel, Duren, Germany) and then stored at −72 °C for analysis. Using Takara Ex-Taq DNA polymerase (Takara Bio, Shiga, Japan) and primer sets (forward: 5′-GGACTACHVGGGTWTCTAAT-3′ and reverse: 5′-GTGCCAGCMGCCGCGGTAA-3′), the 16S ribosomal (rRNA) V4 region from the total isolated genomic DNA was amplified. The amplification was carried out in 30 cycles of 45 s each at 94 °C, 60 s at 55 °C, 90 s at 72 °C, and one cycle of 10 min at 72 °C. Using agarose gel electrophoresis and the QIAquick gel extraction kit (Qiagen, Valencia, CA, US), respectively, the amplicons were separated and purified. Paired-end sequence reads from the DNA library's sequencing on the Illumina MiSeq platform were then quality-trimmed and de-multiplexed using custom Perl scripts. Quantitative Insights Into Microbial Ecology (QIIME 1.9.1) was used to process and evaluate filtered readings in order to determine the diversity and richness indices of the microbial community [11]. When each read had a 97% sequence identity with the Greengenes 13_8 database, it was nominated as an Operating Taxonomic Unit (OTU). The OTUs were then normalized to 25,000 reads per sample by single rarefaction. Principal Coordinate Analysis (PCoA) was consequently drawn based on UniFrac distances as visualized with EMPeror Software [11].

### Sample collection and analyses of non-sanitary environment and intestinal digesta bacterial population

Twenty-five places were sampled using pre-moistened sponge swabs and 10 mL buffered peptone water (Becton, Dickinson and Co., Franklin Lakes, NJ, US) from feeders, cage walls and floors. Before being processed further, samples were kept at 2 °C for 24 h after being delivered to the lab. Samples were homogenized by putting them in a masticator (Masticator Silver, IUL S.A., Barcelona, Spain) after being diluted with 25 mL of Buffered Peptone Water. Swab samples were further diluted in peptone physiological salt water (Becton, Dickinson and Co., Franklin Lakes, NJ, US) before plating in order to obtain countable results on the chosen agar media, including Slanetz-and-Bartley (Oxoid CM0377, Basingstoke, Hampshire, England) for *Enterococcus* spp., tryptose-sulfitecycloserine (TSC) agar (Oxoid, Basingstoke, Hampshire, England) for *Clostridium* spp., and Biorad 356–4024 (Marnes-la-Coquettes, France) for rapid *E. coli*, and violet red bile agar (Merck Co., Ltd., Germany) for total coliforms. To acquire samples of the digestive tract for microbial population study, puncturing the duodenum, jejunum, ileum and cecum was used [1, 10]. In brief, 9 mL of sterile peptone phosphate-buffered saline (0.1%) was completely mixed with 1 g of digesta sample from each part of the gut, including the duodenum, jejunum, ileum and cecum. The following methods were employed to identify the *Lactobacillus* spp. (MRS agar + 0.200 g/L NaN_3_ + 0.500 g/L *L*‐cystine hydrochloride monohydrate, 48 h incubation at 37 °C; Difco Laboratories, Detroit), *Clostridium* spp. (TSC agar; 48 h incubation at 37 °C; Oxoid, Hampshire, UK), and coliforms (violet red bile agar, 24 h incubation at 37 °C; Merck Co., Ltd., Germany). Prior to statistical analysis, the bacterial concentration was determined by averaging duplicate plates and expressed as (log, pfu/kg).

### Statistical analyses

The MIXED procedure of the SAS software (version 9.4, SAS Institute, Cary, NC, US) was used to conduct statistical analyses for parametric factors on growth performance, culture-based intestinal digesta, and intestinal morphology. The nonparametric Kruskal–Wallis test was used to examine the significant differences between the groups for nonparametric variables, such as taxonomic comparisons from 16S rDNA sequencing data. A correction for multiple comparisons was made using the Bonferroni correction test. The QIIME pipeline (alpha diversity.py) used rarefaction with 10 iterations to construct the alpha diversity indices. The R software was used to perform a one-way analysis of variance along with Tukey's post-hoc test and Kruskal–Wallis test (version 4.0.2). *P* < 0.01 and *P* < 0.05 were considered statistically significant. The influences on the microbial community at various sample stages were determined using Adonis statistical tests using QIIME, with 999 permutations, and PCoA was assessed based on unweighted and weighted UniFrac distances.

## Results

### Growth performance

Pigs in the BP and ZnO treatments had significantly higher final BW, ADG and G/F compared with the NC (Table 1). There was no difference between the BP and ZO for any growth parameters. The feed intake was not affected by the treatments.

**Table 1:** Effects of bacteriophage cocktail or ZnO on growth performance of weaned pigs subjected to non-sanitary environment

Treatment	PC	NC	BP	ZO	SEM	*P*-value
Sanitary environment	+	-	-	-		
ADG, g	228.4^ab^	196.9^b^	262.3^a^	277.6^a^	8.47	0.001
ADFI, g	397.5	400.0	412.1	424.0	6.53	0.471
G/F	0.57^ab^	0.50^b^	0.63^a^	0.65^a^	0.02	0.001

*PC* Sanitary environment, *NC* Non-sanitary environment, *BP* Non-sanitary environment plus bacteriophage cocktail, *ZO* Non-sanitary environment plus 2,500 mg/kg ZnO, *ADG* Average daily gain, *ADFI* Average daily feed intake, *G/F* Gain to feed ratio

^a,b^Mean values within a row with unlike superscript letters were significantly different (*P* < 0.05)

### Fecal score

There was no difference in pigs fecal score at d 1 (Table 2). Among the non-sanitary treatments, the ZO treatment showed the lowest fecal score at d 3. The fecal score of pigs in the NC treatment was the highest at d 5, 8, 11, and 14. There were no differences in the fecal score between the BP and ZO treatments at d 5, 8, 11 and 14.

**Table 2:** Effects of bacteriophage cocktail or ZnO on fecal score of weaned pigs subjected to non-sanitary environment

Treatment	PC	NC	BP	ZO	SEM	*P*-value
Sanitary environment	+	-	-	-		
Fecal score						
d 1	2.98	2.91	2.81	2.72	0.11	0.441
d 3	2.51^b^	2.82^a^	2.11^ab^	1.77^c^	0.15	0.006
d 5	1.89^b^	2.66^a^	1.91^b^	1.98^b^	0.17	0.011
d 8	1.81^b^	2.39^a^	1.70^b^	1.74^b^	0.12	0.014
d 11	1.89^b^	2.27^a^	1.42^c^	1.35^c^	0.19	0.009
d 14	1.78^b^	2.31^a^	1.19^c^	1.26^c^	0.16	0.008

*PC* Sanitary environment, *NC* Non-sanitary environment, *BP* Non-sanitary environment plus bacteriophage cocktail, *ZO* Non-sanitary environment plus 2,500 mg/kg ZnO

^a-c^Mean values within a row with unlike superscript letters were significantly different (*P* < 0.05)

### Inflammatory response and intestinal permeability indicators

**Fig. 1:**
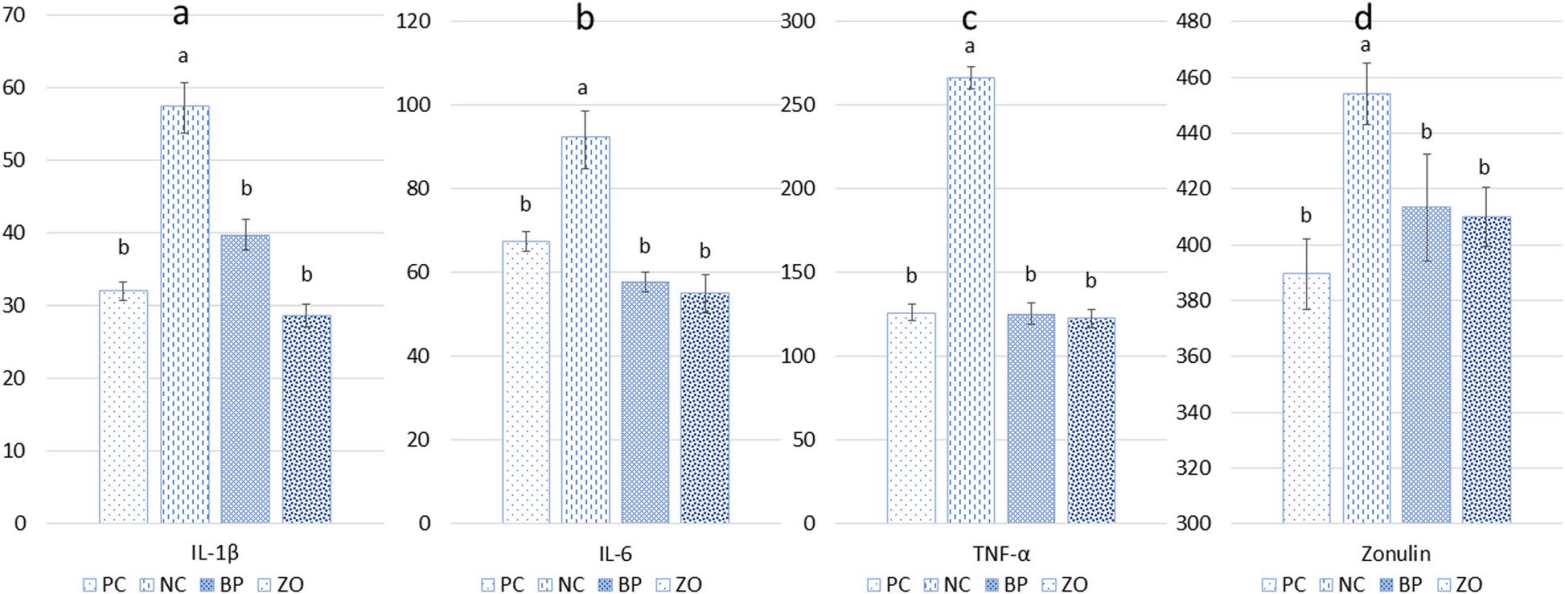
Effects of bacteriophage cocktail or ZnO on blood cytokines and zonulin concentration of weaned pigs subjected to non-sanitary environment. PC, sanitary environment; NC, non-sanitary environment; BP, non-sanitary environment plus bacteriophage cocktail; ZO, non-sanitary environment plus 2,500 mg/kg ZnO. ^a,b^Different letters indicate significant difference between groups (*P* < 0.05)

**Fig. 2:**
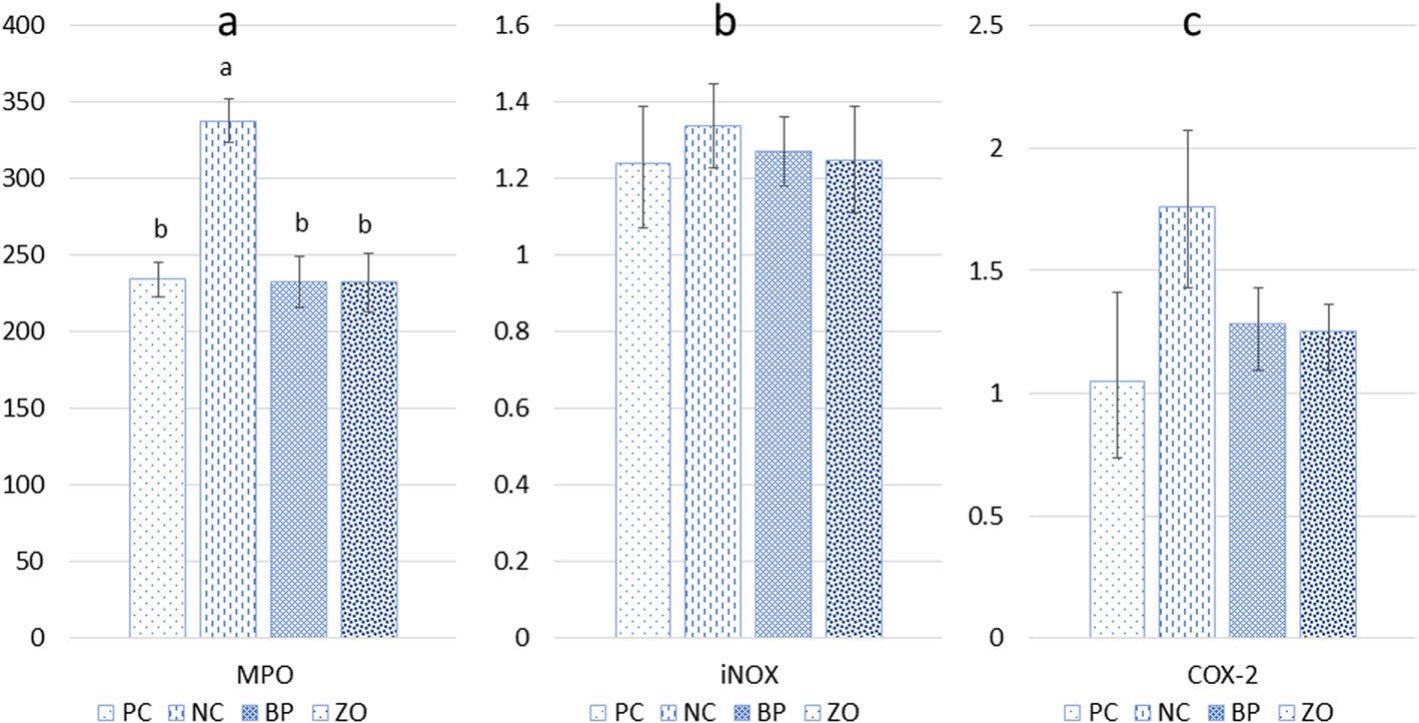
Effects of bacteriophage cocktail or ZnO on inflammatory enzymes in the jejunum of weaned pigs subjected to non-sanitary environment. PC, sanitary environment; NC, non-sanitary environment; BP, non-sanitary environment plus bacteriophage cocktail; ZO, non-sanitary environment plus 2,500 mg/kg ZnO. ^a,b^Different letters indicate significant difference between groups (*P* < 0.05)

The NC treatment showed the highest concentration of inflammatory cytokines including IL-1β, IL-6 and TNF-α in the plasma (Fig. 1). There was no difference in inflammatory cytokines concentrations among PC, BP and ZO treatments. Among the non-sanitary treatments, the NC treatment showed a higher concentration of zonulin in the plasma compared with the BP and ZO. There were no differences in the concentration of zonulin between the BP or ZO treatments and the PC. The administration of BP and ZO showed lower MPO concentrations compared with the NC (Fig. 2). The iNOS and COX-2 were unaffected in the PC and non-sanitary treatments.

### Antioxidant status and serum alkaline phosphatase and aminotransferase

**Fig. 3:**
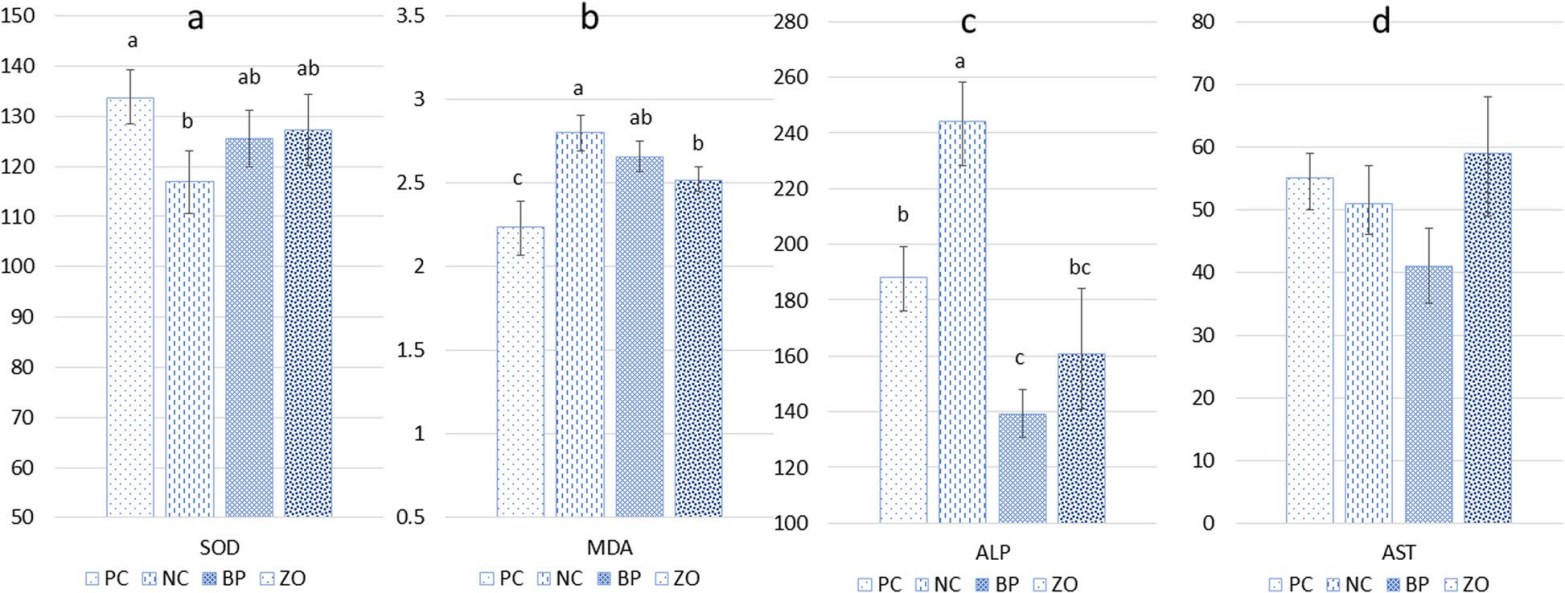
Effects of bacteriophage cocktail or ZnO on antioxidant status and liver-injury enzymes in the jejunum of weaned pigs subjected to non-sanitary environment. PC, sanitary environment; NC, non-sanitary environment; BP, non-sanitary environment plus bacteriophage cocktail; ZO, non-sanitary environment plus 2,500 mg/kg ZnO. ^a-c^Different letters indicate significant difference between groups (*P* < 0.05)

The NC treatment showed a lower concentration of SOD in serum compared with the PC (Fig. 3). However, there was no difference between the non-sanitary treatments (NC, BP and ZO). The PC treatment showed the lowest concentration of MDA. Among the non-sanitary treatments, the NC treatment showed a higher concentration of MDA compared with the ZO, but no difference was detected between the NC and BP treatments. There were no differences in plasma concentration of MDA between the BP and ZO. The NC treatment showed the highest concentration of ALP in the serum, while the ALP concentration in the BP treatment was lower than the PC. There was no difference in concentration of AST in the serum.

### Short-chain fatty acids

There were no differences in fecal concentration of acetic acid, propionic acid, isobutyric acid and total SCFA among treatments (Table 3). The PC treatment showed a lower concentration of butyric acid in the feces compared with the BP treatment. Among the non-sanitary treatments, there was no difference in concentration of butyric acid, however, there was a tendency for higher butyric acid in the BP compared with the NC.

**Table 3:** Effects of bacteriophage cocktail or ZnO on inflammation related enzyme of weaned pigs subjected to non-sanitary environment, mg/g

Treatment	PC	NC	BP	ZO	SEM	*P*-value
Sanitary environment	+	-	-	-		
Acetic acid	3.75	4.25	5.02	4.64	0.61	0.208
Propionic acid	2.72	2.59	2.93	2.07	0.40	0.190
Butyric acid	1.02^b^	1.44^ab^	2.35^a^	2.02^ab^	0.41	0.012
Isobutyric acid	0.14	0.22	0.20	0.27	0.07	0.359
Total short-chain fatty acids	7.61	8.49	10.46	8.99	1.23	0.150

*PC* Sanitary environment, *NC* Non-sanitary environment, *BP* Non-sanitary environment plus bacteriophage cocktail, *ZO* Non-sanitary environment plus 2,500 mg/kg ZnO

^a,b^Mean values within a row with unlike superscript letters were significantly different (*P* < 0.05)

### Morphology

As shown in Table 4, there was no difference in VH of the duodenum, jejunum and ileum between the PC and non-sanitary treatments. Among non-sanitary treatments, the VH in the duodenum was greater in the BP and ZO compared with the NC. Moreover, the VH in the jejunum was increased in the ZO compared with the NC, however, there was no difference between BP and ZO treatments. There was a tendency for higher VH in the ileum of the BP and ZO treatments compared with the NC. There was no difference in crypt depth and VH/CD in the duodenum, jejunum and ileum.

**Table 4:** Effects of bacteriophage cocktail or ZnO on small intestine morphology of weaned pigs subjected to non-sanitary environment, µm

Treatment	PC	NC	BP	ZO	SEM	*P*-value
Sanitary environment	+	-	-	-		
Villus height (VH)						
Duodenum	520^ab^	493^b^	532^a^	539^a^	11.01	0.002
Jejunum	552^ab^	534^b^	561^ab^	568^a^	11.54	0.042
Ileum	437	418	446	455	13.80	0.084
Crypt depth (CD)						
Duodenum	323	344	335	360	15.75	0.160
Jejunum	321	300	304	309	12.86	0.393
Ileum	252	261	274	251	18.65	0.596
VH/CD						
Duodenum	1.61	1.45	1.59	1.51	0.77	0.166
Jejunum	1.73	1.79	1.85	1.84	0.78	0.404
Ileum	1.75	1.63	1.65	1.84	0.48	0.386

*PC* Sanitary environment, *NC* Non-sanitary environment, *BP* Non-sanitary environment plus bacteriophage cocktail, *ZO* Non-sanitary environment plus 2,500 mg/kg ZnO

^a,b^Mean values within a row with unlike superscript letters were significantly different (*P* < 0.05)

### Non-sanitary environment and intestinal microbiota structure

**Fig. 4:**
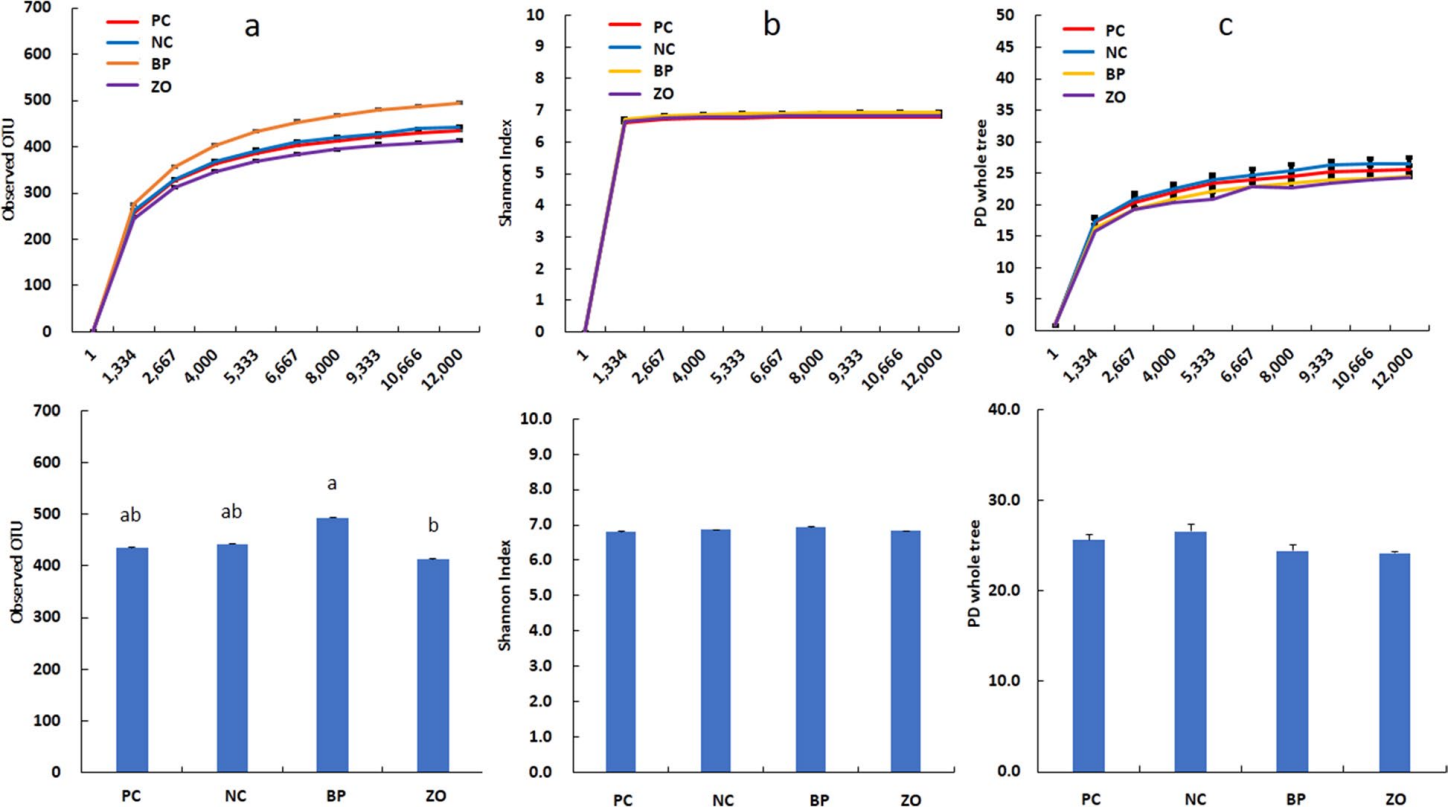
Alpha diversity of weaned pigs gut microbiota including total observed OTU (**a**), Shannon index (**b**), and phylogenetic diversity (PD) whole tree (**c**). PC, sanitary environment; NC, non-sanitary environment; BP, non-sanitary environment plus bacteriophage cocktail; ZO, non-sanitary environment plus 2,500 mg/kg ZnO. ^a,b^Different letters indicate significant difference between groups (*P* < 0.05)

**Fig. 5:**
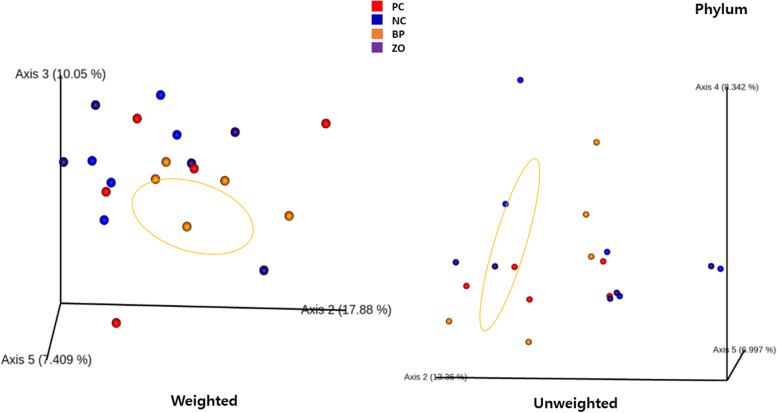
Evaluation of beta diversity patterns of fecal microbial diversity in weaned pigs according to principal coordinate analysis of unweighted and Weighted Unifrac. PC, sanitary environment; NC, non-sanitary environment; BP, non-sanitary environment plus bacteriophage cocktail; ZO, non-sanitary environment plus 2,500 mg/kg ZnO

**Fig. 6:**
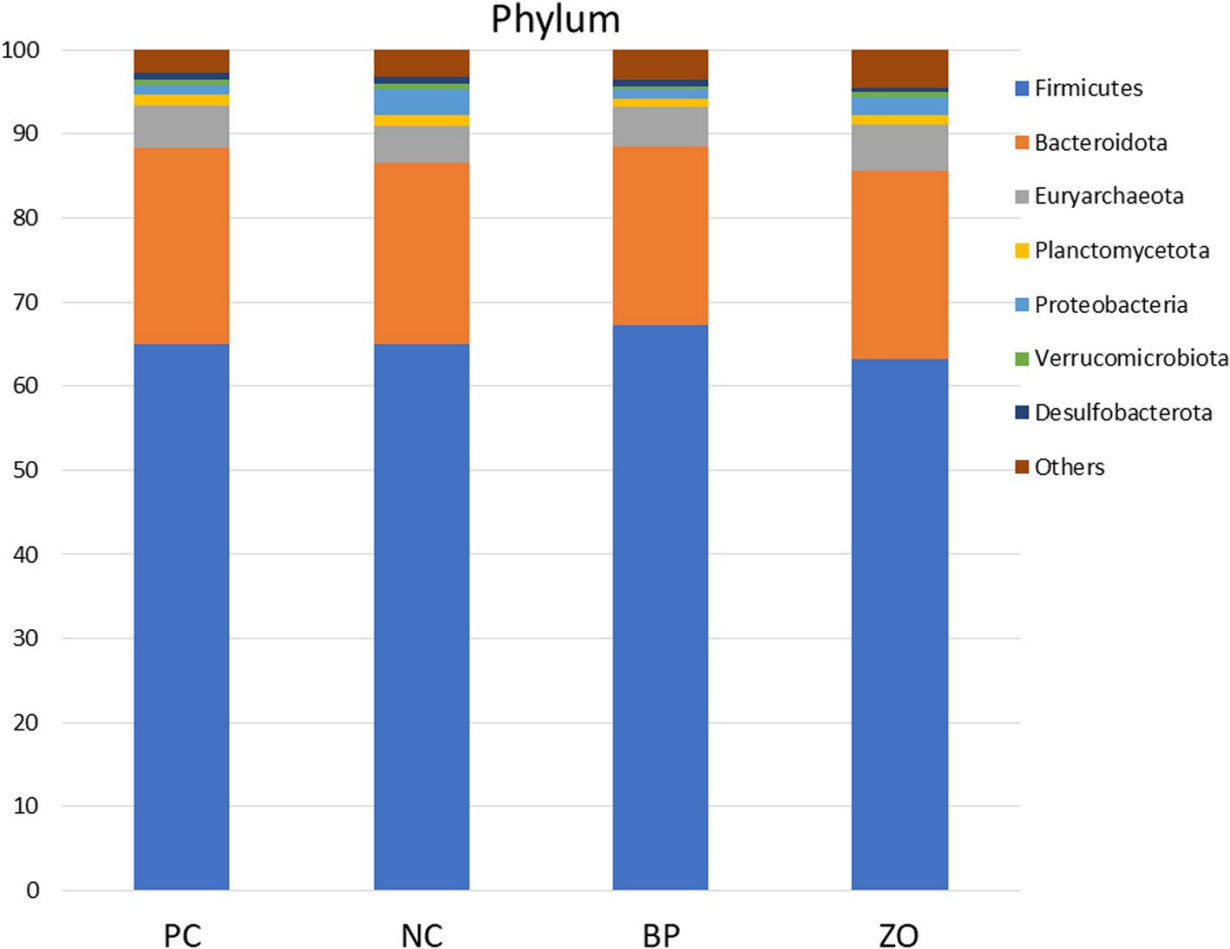
Weaned piglets fecal bacterial community structure at the phylum level was identified by 16S rRNA gene analysis. PC, sanitary environment; NC, non-sanitary environment; BP, non-sanitary environment plus bacteriophage cocktail; ZO, non-sanitary environment plus 2,500 mg/kg ZnO

**Fig. 7:**
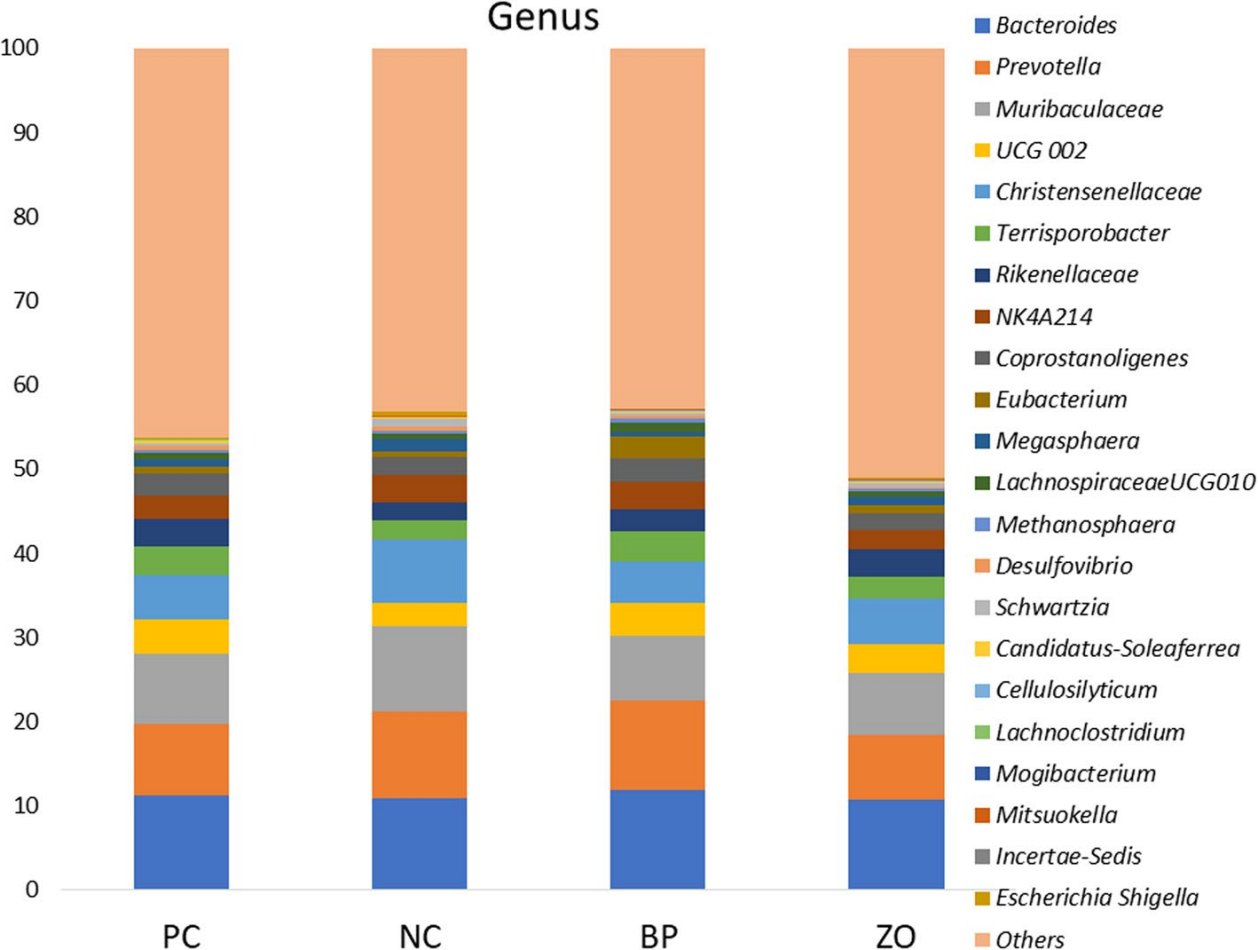
Weaned piglets fecal bacterial community structure at the genus level was identified by 16S rRNA gene analysis. PC, sanitary environment; NC, non-sanitary environment; BP, non-sanitary environment plus bacteriophage cocktail; ZO, non-sanitary environment plus 2,500 mg/kg ZnO

**Fig. 8:**
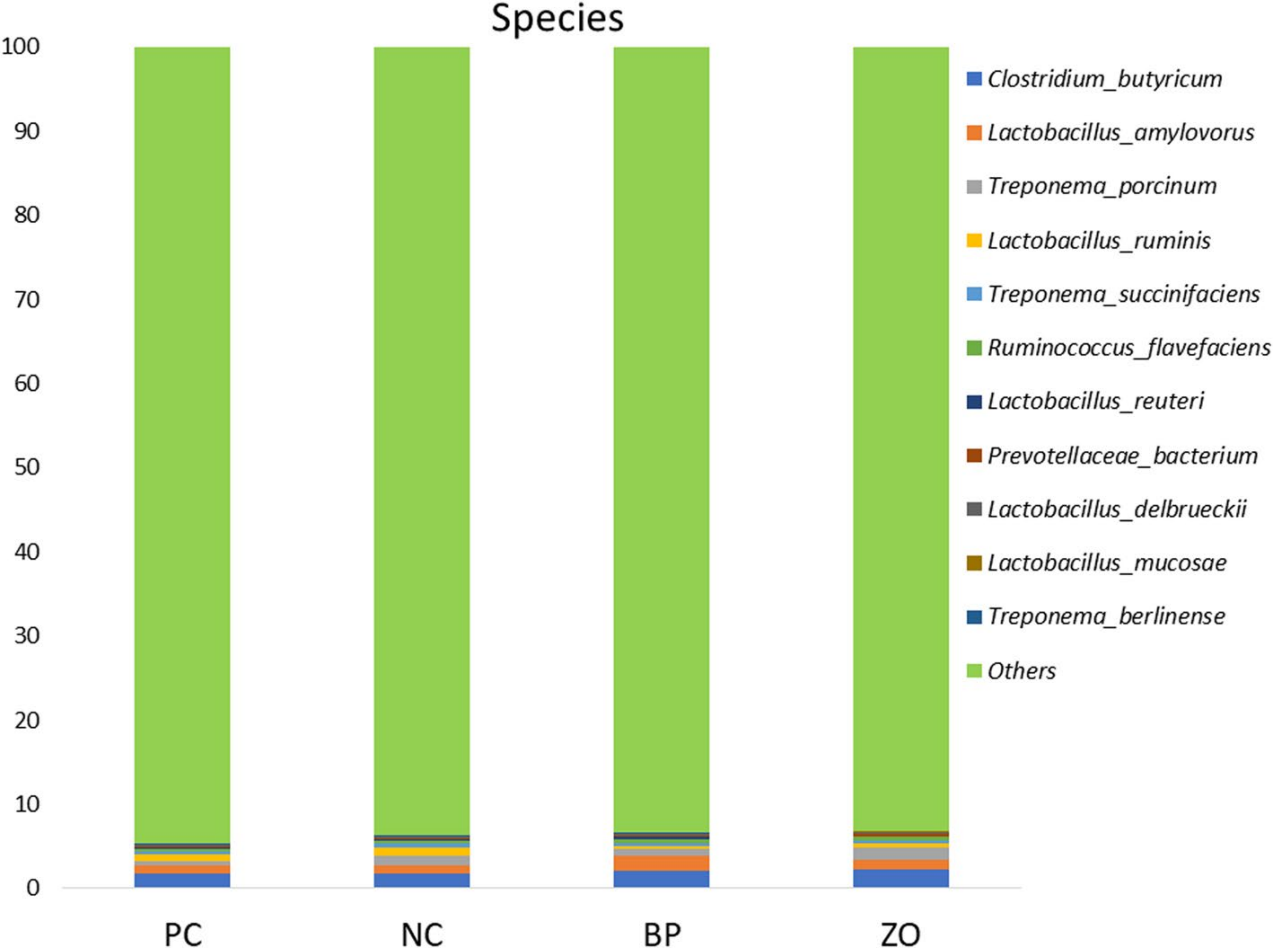
Weaned piglets fecal bacterial community structure at the species level was identified by 16S rRNA gene analysis. PC, sanitary environment; NC, non-sanitary environment; BP, non-sanitary environment plus bacteriophage cocktail; ZO, non-sanitary environment plus 2,500 mg/kg ZnO

Before starting the experiment, the swab samples from the cages showed positive result in all the samples for *E. coli*, coliforms, *Clostridium* spp., and *Enterococcus* spp. An average of 26,000 16S rRNA sequence reads was conducted. The observed OTUs number (± SE) was 434.2 (± 14.3) for the PC, 441.9 (± 6.7) for the NC, and 493.6 (± 12.55) for the BP, and 412.7 (± 17.4) for the ZO treatment (Fig. 4a). The observed OTU showed a greater value in the BP treatment compared with the ZO. The α-diversity, calculated as Shannon indices, did not show any differences across groups (Fig. 4b). The phylogenetic diversity whole tree showed no difference among the treatments (Fig. 4c). The unweighted UniFrac distance analysis using the Adonis test (Fig. 5) revealed no differences between the treatments. Similar comparisons between weighted and unweighted UniFrac distances revealed no differences between the treatments. Bacterial sequences were divided into nineteen phyla, with Firmicutes being the majority, followed by Bacteroidota, as well as Euryarchaeota, Planctomycetota, Proteobacteria, Spirochaetota, Verrucomicrobia and Desulfobacterota appearing in the minor positions. Bacterial taxa of Firmicutes, Bacteroidota, Euryarchaeota, Planctomycetota, Spirochaetota, Verrucomicrobia, and Desulfobacterota did not differ between treatment groups and time points (Fig. 6 and Table 5). The only significant difference occurred for Proteobacteria phylum which was significantly lower in the BP and PC treatments compared with the NC. At the 97% similarity level, a total of 432 genera were detected. At the genus level, six dominant genera, *Bacteroides* (11.17%), *Prevotella* (9.32%), *Muribaculaceae* (8.35%), *Christensenellaceae* R-7 group (5.75%), *UCG*-002 (3.59%), and *Terrisporobacter* (3.01%), were detected in the fecal microbiota of weaned pigs with no significant differences between the treatments, whereas the relative abundance of *Rikenellaceae* RC9 gut group was higher in the PC and ZO treatments compared with the NC (Fig. 7 and Table 6). Although the differences in the abundance of *NK*4A214 group and *Coprostanoligenes* group did not differ, the highest relative abundance of *Eubacterium* was recorded in the BP treatment. The abundance of *Megasphaera* and *Schwartzia* was higher in the NC pigs compared with the BP piglets. Compared to the NC, the abundance of *Lachnospiraceae* UCG-010 was higher in the BP, and the abundance of *Desulfovibrio* was lower in the supplemented treatments (BP and ZO) compared with non-supplemented (NC and PC). The abundance of *Cellulosilyticum* genera was higher in the BP and ZO treatments rather than in the NC, whereas a higher abundance of *Incertae Sedis* was detected in the BP compared with the ZO. The piglets in the NC treatment had the highest abundance of *Escherichia-Shigella*, followed by the PC and ZO treatments. The relative species abundance showed no difference between the treatments for *Clostridium butyricum, Lactobacillus amylovorus, Treponema porcinum, Treponema succinifaciens, Ruminococcus flavefaciens, Lactobacillus reuteri, Prevotellaceae bacterium, Lactobacillus delbrueckii, Lactobacillus mucosae,* and *Treponema berlinense* (Fig. 8 and Table 7)*.* The only change was detected for *Lactobacillus ruminis* which was significantly higher in the NC treatment compared with the BP. The culture-based intestinal microbiota showed a significant increase of *Lactobacillus* spp. in pigs in the BP, ZO, and PC treatments (Table 8). The number of *Bifidobacterium* spp. was higher in the BP compared with the PC and ZO. Pigs in the NC treatment had the highest population of *Clostridium* spp. The population of coliforms was the lowest in the BP treatment.

**Table 5:** Effects of bacteriophage cocktail or ZnO on the relative phyla abundance of weaned pigs subjected to non-sanitary environment

Treatment	PC	NC	BP	ZO	SEM	*P*-value
Sanitary environment	+	-	-	-		
Firmicutes	65.00	65.04	67.19	63.24	3.36	0.439
Bacteroidota	23.29	21.48	21.34	22.35	1.31	0.481
Euryarchaeota	5.10	4.44	4.71	5.55	0.56	0.509
Planctomycetota	1.23	1.26	1.04	1.05	0.16	0.544
Proteobacteria	1.13^b^	3.12^a^	0.99^b^	2.16^ab^	0.64	0.041
Spirochaetota	2.13	2.52	2.87	3.87	0.92	0.167
Verrucomicrobiota	0.66	0.62	0.46	0.63	0.07	0.253
Desulfobacterota	0.87	0.76	0.74	0.50	0.14	0.083

*PC* Sanitary environment, *NC* Non-sanitary environment, *BP* Non-sanitary environment plus bacteriophage cocktail, *ZO* Non-sanitary environment plus 2,500 mg/kg ZnO

^a,b^Mean values within a row with unlike superscript letters were significantly different (*P* < 0.05)

**Table 6:** Effects of bacteriophage cocktail or ZnO on the relative genera abundance of weaned pigs subjected to non-sanitary environment

Treatment	PC	NC	BP	ZO	SEM	*P*-value
Sanitary environment	+	-	-	-		
*Muribaculaceae*	8.20	10.14	7.66	7.39	1.02	0.096
*UCG-002*	4.12	2.80	3.94	3.50	0.48	0.068
*Christensenellaceae_R-7_group*	5.29	7.52	4.88	5.31	0.98	0.073
*Terrisporobacter*	3.42	2.28	3.66	2.69	0.58	0.069
*Rikenellaceae_RC9_gut_group*	3.28^a^	2.05^b^	2.60^ab^	3.24^a^	0.37	0.031
*NK4A214_group*	2.76	3.21	3.19	2.32	0.38	0.053
*Coprostanoligenes_group*	2.57	2.15	2.75	1.94	0.29	0.075
*Eubacterium*	0.89^b^	0.76^b^	2.72^a^	1.01^b^	0.18	0.009
*Megasphaera*	0.85^ab^	1.44^a^	0.35^b^	0.88^ab^	0.33	0.035
*Lachnospiraceae_UCG010*	0.81^ab^	0.68^b^	1.21^a^	0.66^b^	0.26	0.031
*Methanosphaera*	0.32	0.27	0.58	0.31	0.13	0.077
*Desulfovibrio*	0.36^a^	0.49^a^	0.19^b^	0.17^b^	0.06	0.011
*Schwartzia*	0.48^b^	0.93^a^	0.37^b^	0.61^ab^	0.14	0.010
*Candidatus_Soleaferrea*	0.22	0.15	0.26	0.14	0.33	0.056
*Cellulosilyticum*	0.081^ab^	0.032^b^	0.100^a^	0.101^a^	0.02	0.018
*Lachnoclostridium*	0.076	0.055	0.046	0.067	0.014	0.097
*Mogibacterium*	0.030	0.032	0.014	0.026	0.08	0.079
*Mitsuokella*	0.022	0.077	0.031	0.133	0.49	0.082
*Incertae_Sedis*	0.048^ab^	0.045^ab^	0.060^a^	0.019^b^	0.14	0.046
*Escherichia-Shigella*	0.16^b^	0.50^a^	0.04^c^	0.19^b^	0.05	0.006

*PC* Sanitary environment, *NC* Non-sanitary environment, *BP* Non-sanitary environment plus bacteriophage cocktail, *ZO* Non-sanitary environment plus 2,500 mg/kg ZnO

^a,b^Mean values within a row with unlike superscript letters were significantly different (*P* < 0.05)

**Table 7:** Effects of bacteriophage cocktail or ZnO on the relative species abundance of weaned pigs subjected to non-sanitary environment

Treatment	PC	NC	BP	ZO	SEM	*P*-value
Sanitary environment	+	-	-	-		
*Clostridium_butyricum*	1.78	1.75	2.06	2.32	0.29	0.191
*Lactobacillus_amylovorus*	0.92	0.95	1.76	1.10	0.41	0.141
*Treponema_porcinum*	0.54	1.14	0.83	1.37	0.49	0.241
*Lactobacillus_ruminis*	0.77^ab^	0.98^a^	0.31^b^	0.59^ab^	0.22	0.030
*Treponema_succinifaciens*	0.42	0.53	0.33	0.28	0.17	0.343
*Ruminococcus_flavefaciens*	0.33	0.29	0.54	0.44	0.21	0.540
*Lactobacillus_reuteri*	0.17	0.25	0.33	0.15	0.08	0.152
*Prevotellaceae_bacterium*	0.15	0.13	0.20	0.32	0.07	0.098
*Lactobacillus_delbrueckii*	0.08	0.14	0.13	0.11	0.33	0.270
*Lactobacillus_mucosae*	0.07	0.08	0.04	0.10	0.25	0.154
*Treponema_berlinense*	0.09	0.08	0.12	0.08	0.31	0.473

*PC* Sanitary environment, *NC* non-sanitary environment, *BP* Non-sanitary environment plus bacteriophage cocktail, *ZO* Non-sanitary environment plus 2,500 mg/kg ZnO

^a,b^Mean values within a row with unlike superscript letters were significantly different (*P* < 0.05)

**Table 8:** Effects of bacteriophage cocktail or ZnO on fecal microbiota of weaned pigs subjected to non-sanitary environment

Treatment	PC	NC	BP	ZO	SEM	*P*-value
Sanitary environment	+	-	-	-		
*Lactobacillus* spp.	8.21^ab^	7.86^b^	8.69^a^	8.36^a^	0.15	0.029
*Bifidobacterium* spp.	8.01^b^	8.43^ab^	8.62^a^	8.03^b^	0.11	0.021
*Clostridium* spp.	6.12^b^	6.66^a^	6.01^b^	6.16^b^	0.111	0.046
Coliforms	6.71^a^	6.82^a^	5.11^b^	6.41^a^	0.182	< 0.001

*PC* Sanitary environment, *NC* Non-sanitary environment, *BP* Non-sanitary environment plus bacteriophage cocktail, *ZO* Non-sanitary environment plus 2,500 mg/kg ZnO

^ac^Mean values within a row with unlike superscript letters were significantly different (*P* < 0.05)

## Discussion

It has been shown that bacteriophages can have a growth-promoting role in pig growth [9, 10, 17, 20], and their anti-pathogenic role can be even more highlighted if pigs raise in non-sanitary conditions. The impact of bacteriophages on pigs performance in a non-sanitary condition has not been well investigated. Animals raised in unsanitary conditions may exhibit decreased growth as a result of intestinal malfunction caused by bacterial challenges. In the current study, we demonstrated that the non-sanitary environment had a detrimental impact on the growth performance of weaned pigs, as indicated by decreased ADG, and final BW along with a reduction of G:F. In general, *E. coli* causes a wide range of production issues, including intestine disturbance, diarrhea and subsequent adverse effect on growth performance in weaned pigs [3]. ZnO had been known as an effective anti-diarrhea agent, and an increase in ADG of weaned pigs had always been observed upon administration of ZnO during the weaning period [9, 15, 19]. In this study, dietary bacteriophage showed a comparable growth performance in comparison with the ZO treatment. Both ZO and BP treatments showed anti-inflammatory and antioxidant effects by reducing IL-1β, IL-6, TNF-α and MPO concentration, which can be responsible for the improved ADG, G:F, and higher VH in the duodenum and jejunum.

The immune system can immediately be affected by bacteriophages during sepsis, when their lytic function can lower the bacterial load. The inflammatory reaction caused by pathogens may be partially buffered due to bacteriophages immunomodulatory abilities [29]. The authors observed decreased secretion of pro-inflammatory cytokines (IL-1β, TNF-α and IL-6) in animals treated with bacteriophage and ZnO, but no change in concentrations of anti-inflammatory cytokine (IL-10), suggesting that bacteriophage and ZnO has a protective effect by reducing the pro-inflammatory response. These cytokines are released during T-helper 2 responses from tool like receptor-4 stimulation, which is related to bacterial LPS production [2]. Although we did not evaluate the levels of circulating LPS, it was well documented that the increased aspartate AST and ALP in whole blood samples can be an indicator of high circulating LPS [30]. Therefore, it is likely that the bacteriophage therapy reduced the amount of circulating LPS, which may be responsible for the reduction in the secretion of IL-1β, TNF-α and IL-6 pro-inflammatory cytokines. The anti-inflammatory effects of bacteriophages shown in the current study have also been confirmed by previous reports in various experimental designs [1, 6, 17]. In pigs treated with bacteriophage, lower levels of the pro-inflammatory cytokine IL-1β and higher levels of the anti-inflammatory cytokines IL-4 and IL-10 were reported [8], indicating that bacteriophage has a protective impact by lowering the pro-inflammatory response. By lowering the pro-inflammatory response, bacteriophages may have a protective impact against diarrheal infection. The BP treatment showed the highest *Eubacterium* population. The reduction of *Eubacterium* has been linked to a number of inflammatory gastrointestinal diseases [31]. Also, there was a positive relationship between the *Escherichia-Shigella* and sulfate-reducing bacteria *Desulfovibrio*. The effect of sulfate-reducing bacteria on the inflammation in the intestine is still not fully known, however, it is well acknowledged that the generation of hydrogen sulfide may have inflammatory and cytotoxic consequences in the gut that may compromise epithelial barrier integrity [31]. In addition, after the exposure of pigs to a non-sanitary environment, we discovered intestinal tight junction damage, which was evident by the increase of zonulin in the serum. Recent research has shown that administering bacteriophage to pigs could increase the expression of intestinal tight junction proteins [17, 21] and reduce inflammatory cytokines [21]. The tight junction proteins form a paracellular permeability barrier and a fence to stop the translocation of large molecules [8]. This could result in the challenged pigs having higher serum endotoxins and inflammatory factors.

The first line of immune system cellular defense is formed by neutrophils [18, 32]. However, excessive neutrophil activation leads to the production of toxic mediators including MPO, which encourages tissue damage and inflammatory reactions [8, 33]. In the BP and ZO groups, the inflammatory response, as measured by the density of MPO-positive cells in the jejunum, showed a decrease within 14 d of being exposed to a non-sanitary environment. The anti-*E. coli* effect of bacteriophage may be associated with reduced MPO activity and consequently reduced inflammatory responses. Intestinal microbiota affects MPO production [34]. In general, the generation of glutathione and the reduction of MDA levels and inflammatory mediators such as MPO, TNF-α, IL-1β and interferon-γ are as part of the antioxidant mechanism and associated pathways [32, 35]. MPO, an organic natural defense for immune response, in jejunum is an important marker of the increase of intestinal inflammation [32, 33]. Probiotic supernatants manufactured from *E. coli* Nissle 1917 and *Lactobacillus fermentum* BR11 decreased the histological scores and MPO activity levels in the jejunum of rats given 5-fluorouracil toxin [36]. In agreement, Li et al. [33] showed that the exposure of pigs to a polluted environment shows a positive correlation between MPO concentration and *Pseudomonas* bacteria. In conclusion, the current study showed that non-sanitary conditions increased levels of MPO, suggesting that pathogenic colonization led to inflammatory damage in the jejunum. However, with BP and ZO supplementations, the jejunum's inflammatory damage could be mitigated to a certain extent. Another study also demonstrated that a pharmacological dose of ZnO has potent anti-inflammatory, antioxidant, and protective effects by inhibiting polymorphonuclear leukocyte population activity, as well as by quantitatively reducing MPO and MDA [14, 18], and pro-inflammatory mediators like TNF-α and IL-1β, all of which are crucial in oxidative stress [37]. Moreover, in the current study, it was found that bacteriophage and ZnO reduced COX-2 release. Although there are about 60% similarities between different COX structures, COX-2 possesses higher affinity and sensitivity to conformational variations in the active site rather than other cyclooxygenases. COX-2 plays a crucial role in inflammatory responses by the release of arachidonic acid and producing eicosanoids such as prostaglandins that are engaged in different immunological and inflammatory responses [13]. Several studies have reported the adverse effect of dysbiosis and pathogenic microbial agents on the overexpression of COX-2 in mucositis models in the intestine [13]. Then, the tendency for lower anti-inflammatory influence of bacteriophage on jejunal mucosa can be associated with its anti-pathogenic properties and the possibility of interactions with COX-2 and COX-2 pathways.

The hypothesis of the protective action of bacteriophage on inflammatory reactions has been supported by studies conducted in situations where oxidative stress played a significant role in initiating and maintaining tissue damage. Oxidative stress is caused by an imbalance between the antioxidant systems and oxidative species carried on by the inflammatory responses. It is identified by the generation of free radicals, reactive nitrogen and oxygen species. The natural physiological process is impaired by these reactive species, which further damages structural and/or functional components. In the current study, bacteriophage administration inhibited pigs from oxidative stress and reverses the rise in MDA levels in pigs exposed to a non-sanatory environment. Numerous research reported the antioxidant effect of bacteriophages through the manipulation of microbiota [10, 20, 21, 25, 29]. According to our findings, a non-sanitary environment reduced the antioxidant capacity by decreasing the concentration of SOD, which increased the levels of serum MDA. However, The BP and ZO treatments alleviated these negative effects. ZnO possesses strong antioxidant properties on its own [9, 10, 15, 19], which may help to explain how ZnO works as an antioxidant in a contaminated environment.

The insignificant difference in total SCFA production could have been due to the low dietary fiber as the main substrate for SCFA production, masking responses among the treatments. Butyric acid was the only SCFA with a significantly higher concentration in the BP treatment compared with the PC. The change in microbiota could be associated with higher butyric acid production in the BP treatment. It is interesting to note the supplementation of bacteriophages in the current study led to an 11-fold reduction of *Escherichia-Shigella* and a threefold increase in amplicon sequence variants that map to taxa in the *Eubacterium* genus. *Eubacterium* spp. are known as butyrate producers [38]. These findings imply the importance of additional investigation of the mechanistic relationships between SCFA production and bacteriophage intake, as well as an examination of the impact of the bacteriophage cocktail under stressful conditions like the weaning period.

Bacteriophage improved VH in the duodenum while reducing *Clostridium* spp. and coliforms in the current investigation, reversing the effects of pathogens on changes in gastrointestinal function. In addition the production of butyric acid and *Eubacterium* spp. bacteria was increased in the BP-treated pigs. Since *Eubacterium* spp. produce butyrate, their role in promoting enterocyte turnover and preserving tight barrier junctions may be significant. In parallel, BP-treated pigs had a considerable impact on their small intestinal permeability. Treatment with BP considerably lowered the release of zonulin. A high concentration of zonulin is a sign of a leaky gut and any reduction of zonulin release is connected to the health of the intestinal mucosa [12]. This observation is remarkable because numerous authors have demonstrated that pathogenic bacterial translocation with the emergence of systemic inflammation might be caused by the loss of intestinal epithelial barrier integrity [26, 39, 40]. The microbiota change, butyric acid production, and zonulin concentration may partially explain the observed villus integrity.

The intestinal mucosa is a complex and dynamic structure that plays a vital role in maintaining the health of the gut by allowing the absorption of nutrients [41], preventing the entry of harmful substances, and regulating the immune response to gut bacteria [42]. Additionally, the mucosa also plays a role in regulating the immune system, by stimulating the production of immune cells and inflammatory mediators, thus keeping the gut ecosystem in balance [39]. A healthy mucosa produces considerable mucin to prevent the adhesion of harmful bacteria and viruses to the gut lining [43]. The interactions between bacteriophages and the bacterial hosts are shaped by the intestinal mucosa. A phage-mediated immunity is produced when bacteriophages contact the mucosal barrier [44]. According to this theory, acquired immunity lyses invasive bacteria in the lowest layers of mucus to kill them while innate immunity preserves commensal bacteria through lysogeny in the top layers of mucus [22]. With bacteriophage treatment, the microbiota did not appear to be altered globally, but some specific microbial community members underwent some significant changes [31]. Importantly, the number of *E. coli* and *Clostridium* spp. the target host for the supplemented bacteriophage was reduced. Additionally, there was a positive or negative correlation between populations of several bacterial taxa and *E. coli*. While it is challenging to determine whether specific ecological interactions were responsible for these relationships. There was a positive correlation between *E. coli* and sulfate-reducing bacteria, such as *Desulfovibrio*. Higher diarrhea and inflammatory responses have been linked to both *Desulfovibrio* and *E. coli* [12]. *Desulfovibrio* is a member of the Gram-negative bacteria in phylum Proteobacteria, which showed the lowest abundance in the BP treatment. The species from the Proteobacteria phylum, in particular, has been reported to be high in pigs with intestinal inflammation [45]. Several physiological theories have been offered to explain how bacteriophages affect intestinal microbiota. Bacteriophage potential to prevent pathogen growth may indirectly influence the modulation of intestinal microbiota in favor of beneficial bacteria [1, 6, 21, 31]. The observed attenuation of diarrhea and improved fecal score may be due to the restoration of the microbiota in a competitive environment for pathogenic colonization. The increased population of beneficial bacteria such as *Lactobacillus* spp. was another theory for the therapeutic benefits of bacteriophage treatment on intestinal microbiota. *Bifidobacterium*, *Lactobacillus*, and *Streptococcus* are responsible for mediating the relaxation of colonic motility and reducing diarrhea incidence [2, 40]. The elimination of pathogens may increase the opportunity for beneficial bacteria proliferation. The role of bacteriophage in reducing the number of *Clostridium* spp. and coliforms and increasing *Bifidobacterium* spp., and *Lactobacillus* spp., in pig feces was confirmed [20]. *Bifidobacterium* is a genus of Gram-positive, anaerobic bacteria that are commonly found in the gut microbiome of pigs and play important roles in maintaining gut health and supporting the overall function of the digestive system [1, 10]. *Bifidobacterium* species are known for their ability to ferment various sugars, including lactose, and produce lactic acid as a primary fermentation product [2, 9]. They also produce other compounds such as acetic acid, ethanol, and carbon dioxide, which can contribute to the overall balance of the gut microbiome, improving gut barrier function, and supporting the immune system [46]. An increased OUT was observed in the BP treatment compared with the ZO. The OTU in microbial ecology shows a group of organisms based on their 16S rRNA gene sequences that indicate a shift in the gut microbiome [11, 23]. This shift could be due to various factors such as diet, antibiotics, stress, or anti-microbial activity [11, 39]. An increase in gut microbiome diversity is usually considered a positive thing, as a diverse microbiome has been associated with better health outcomes [45]. According to the PCoA data, the composition of the bacteria varied between treatments but was more similar in the BP group than in the ZO. In agreement, Mu et al. [47] showed that bacteriophage can increase the alpha-diversity of microbiota in pigs. However, in the current study, the OTU showed some controversial results as the higher population of *Lactobacillus ruminis* was observed in the NC treatment compared with the BP and require further research to evaluate the mode of action of such results.

## Conclusion

In conclusion, based on the microbiota information, our study demonstrated that the population of beneficial bacteria such as *Lactobacillus* was significantly increased in the BP-treated piglets. Moreover, the abundance of coliforms was decreased in the feces, which may emphasize the antimicrobial activity of the bacteriophage cocktail. These normal alterations in the gut microbiota at the weaning period decrease the susceptibility of weaned piglets to pathogenic infections due to greater antioxidant and anti-inflammatory response. The greater VH of the duodenum can be considered as the indicator of the integrated intestine that may provide the potential for higher growth performance after weaning. This achievement may provide greater insight into the importance of intestinal microbiota manipulation during weaning, and the role of bacteriophage as an alternative to ZnO.

## Data Availability

The datasets during and/or analysed during the current study are available from the corresponding author upon reasonable request.

## Abbreviations

ADG: Average daily gain
ALP: Alkaline phosphatase
AST: Aminotransferase
BP: Bacteriophage cocktail
BW: Body weight
CD: Crypt depth
COX-2: Cyclooxygenase-2
F/G: Feed:gain ratio
IL: Interleukin
iNOS: Inducible nitric oxide synthase
LPS: Lipopolysaccharide
MPO: Myeloperoxidase
MDA: Malondialdehyde
OTU: Operating taxonomic unit
QIIME: Quantitative insights into microbial ecology
SCFA: Short chain fatty acids
SOD: Super oxide dismutase
TNF-α: Tumor necrosis factor-α
VH: Villus height
